# Regulation of Vessel Permeability by TRP Channels

**DOI:** 10.3389/fphys.2020.00421

**Published:** 2020-05-05

**Authors:** Tullio Genova, Deborah Gaglioti, Luca Munaron

**Affiliations:** Department of Life Sciences and Systems Biology, University of Turin, Turin, Italy

**Keywords:** TRP, endothelial cell, store-operated Ca^2+^entry channels, permeability, vessel permeability, microvessel, TRPC

## Abstract

The vascular endothelium constitutes a semi-permeable barrier between blood and interstitial fluids. Since an augmented endothelial permeability is often associated to pathological states, understanding the molecular basis for its regulation is a crucial biomedical and clinical challenge. This review focuses on the processes controlling paracellular permeability that is the permeation of fluids between adjacent endothelial cells (ECs). Cytosolic calcium changes are often detected as early events preceding the alteration of the endothelial barrier (EB) function. For this reason, great interest has been devoted in the last decades to unveil the molecular mechanisms underlying calcium fluxes and their functional relationship with vessel permeability. Beyond the dicotomic classification between store-dependent and independent calcium entry at the plasma membrane level, the search for the molecular components of the related calcium-permeable channels revealed a difficult task for intrinsic and technical limitations. The contribution of redundant channel-forming proteins including members of TRP superfamily and Orai1, together with the very complex intracellular modulatory pathways, displays a huge variability among tissues and along the vascular tree. Moreover, calcium-independent events could significantly concur to the regulation of vascular permeability in an intricate and fascinating multifactorial framework.

## Features of the Vascular Endothelium

Vascular endothelium lines the intima of the blood vessels, forming a semi-permeable interface between blood and interstitial fluids. Macromolecules cross this barrier via endo/exocytosis (transcellular permeability), while fluids and small solutes can pass endothelium through the space between adjacent ECs (paracellular permeability) (Komarova and Malik, 2010). The permeability of the endothelial layer is finely modulated in order to properly answer local metabolic demands. When the endothelium loses its barrier function, tissue inflammation occurs (Mehta and Malik, 2006).

Responsible for paracellular permeability regulation are the intercellular contacts between ECs maintained by AJs, and the cellular adhesion to the underlying matrix through FACs (Cioffi and Stevens, 2006; Minshall and Malik, 2006). An enhanced endothelial permeability is the result of loss of contact between adjacent microvascular ECs and weakening of their adhesion to the ECM (Minshall and Malik, 2006). Vessel permeability is under the control of pro-inflammatory, thrombogenic mediators and growth factors binding to selective endothelial receptors. A convergent downstream effect is usually an increase of the cytosolic calcium concentration that precedes the enhancement of endothelial permeability. Indeed, cytosolic Ca^2+^ transients induce the retraction of the cell borders by weakening IEJs and cell-matrix junctions. Thus, while ECs change their morphology from a flattened to a rounded shape, gaps between adjacent ECs are formed, allowing the unrestricted flux of plasma proteins, including albumin, and liquid through IEJs (Lum and Malik, 1994; Mehta and Malik, 2006; Minshall and Malik, 2006).

Transient receptor potential channels play a key role in endothelial calcium fluxes occurring during physiological events and human diseases (Hsu et al., 2007; Watanabe et al., 2008; Banner et al., 2011; Galindo et al., 2018; Tsagareli and Nozadze, 2019). Transient receptor potential channels (TRPs) contribute to the regulation of the EB, but the intracellular mechanisms are still partially elusive and highly variable in tissues as well as along the vascular tree.

Here we discuss the state of the art in the field, highlighting the discrepancies and conflicting evidence.

## Endothelial Permeability Along the Vascular Tree

Since an increased endothelial permeability is considered a hallmark of vessel growth in many diseases (Bates and Harper, 2002), huge effort is devoted to uncover the underlying mechanisms, especially within the microcirculation.

In resting rat lung, the vascular filtration coefficient (*K*_*fc*_), indicative of the liquid flux, is near 42% in microvessels and just 19% in arterial regions (Parker and Yoshikawa, 2002). Similar results were found in canine lung (Mitzner and Robotham, 1979).

Such a striking difference can be ascribed to the density of *fenestrae* (Aird, 2007), together with EC shape, thickness and the protein composition of the IEJs. Tight junctions are well developed in large vessels, which have a conduct function, while they are weakened along capillaries, the canonical site for exchange with the surrounding tissue. Thus, the number and complexity of tight junctions appear to be inversely related to permeability (Aird, 2007). Microvascular ECs also express a greater amount of proteins involved in the interaction with the ECM (Chi et al., 2003), explaining why the ECM contribution to permeability prevails in capillaries (Qiao et al., 1995). In addition to these intrinsic mechanisms, also extrinsic factor contribute: blood flow and the relative mechanical stress is pulsatile in large vessels, while linear within capillaries (Mehta and Malik, 2006; Sukriti et al., 2014). Finally, capillary permeability is strongly influenced by the coverage by pericytes, contractile cells wrapped around ECs (Attwell et al., 2016). Indeed, pericytes contraction reversibly opens endothelial gaps, while their loss irreversibly compromises EB (Edelman et al., 2006). Moreover, pericytes control tight junction expression and alignment (Winkler et al., 2012).

## Calcium Signaling Regulates Endothelial Vessel Permeability

Calcium signaling has a central role in the modulation of both physiological and pathological permeability (Curry, 1992; Bates and Curry, 1997; Kelly et al., 1998; Van Nieuw Amerongen et al., 1998; Bates and Harper, 2002; Minshall and Malik, 2006; De Bock et al., 2012). The intracellular calcium concentration ([Ca^2+^]_*i*_) increases in inflammation, causing a reorganization of endothelial gaps (Moore et al., 1998; Mehta and Malik, 2006).

Calcium signals modulate endothelial permeability partly *via* an ubiquitous mode referred to as SOCE, a calcium influx dictated by the depletion of endoplasmic reticulum (ER) calcium stores. The protein STIM1 is located in ER membranes acting as sensor of Ca^2+^ levels in the lumen: upon ER depletion, it underlies a rearrangement to plasma-membrane-ER junctions, where activates SOCs, that include the pore forming protein Orai1 (Smyth et al., 2010) and members of the TRP channel superfamily (Cheng et al., 2013; Ambudkar et al., 2017).

Inflammatory mediators (e.g., thrombin and histamine) bind to plasma membrane G protein-coupled receptors and trigger InsP3-dependent Ca^2+^ release from ER and the following SOCE. The calcium-mediated phosphorylation of MLC drives the formation of actomyosin contractile units and stress fibers, which exert force on the IEJs, weakening them (Dudek and Garcia, 2001; Sandoval et al., 2001; Birukova et al., 2004). In addition, PKC phosphorylates junctional linking proteins vinculin and talin in IEJs and FACs (Lum and Malik, 1994; Rebecchi and Pentyala, 2000; Rhee, 2001). The disassembly of cell-cell and cell-matrix contacts (PKC-mediated passive cell retraction) and the concomitant establishment of contractile units (MLCK-mediated active cell contraction) lead to ECs rounding as well as the formation of intercellular gaps and permeability enhancement.

Early studies highlighted a variable contribution of calcium signaling to vascular permeability between larger and smaller vessels. Kelly et al. (1998) showed that an increase of [Ca^2+^]_*i*_ enough to promote permeability in rPAECs, failed to exert any effect on rPMECs, initially suggesting an apparent uncoupling of [Ca^2+^]_*i*_ signaling pathways or dominant Ca^2+^-independent mechanisms in microvasculature. *In vitro* permeation studies showed that the phosphodiesterase-4 inhibitor Rolipram inhibits SOC in PAECs while revealing it in PMVECs, with consequent shift of the fluid leakage site from big vessels to the microcirculation. Thus, the intracellular events associated with SOCE appear to be site specific, according to the variability of the response to proinflammatory stimuli (Dudek and Garcia, 2001; Wu et al., 2005).

More recently, nSOCs were proposed as major players in microvascular permeability (Alvarez et al., 2006; Cioffi et al., 2009; Komarova et al., 2017; Phuong et al., 2017). Activated following agonist stimulation and independently of store depletion, nSOCs regulate EB (Mehta and Malik, 2006).

## Trps in Endothelial Permeability

Transient receptor potential channels are a superfamily of ion channels, which regulate the plasma membrane permeability to cations in response to a broad range of stimuli. Importantly, 19 of the 28 mammalian TRP channel isoforms are expressed in vascular ECs. Among them, all of the TRPC; TRPV1, -V2, and -V4; all of the TRPM except -M5; and TRPP1 and -P2 (Yao and Garland, 2005; Kwan et al., 2007).

In the following paragraphs, research works that discuss the role of TRP channels in the regulation of EC permeability will be discussed.

The involvement of TRPs in EB regulation is well established (Groschner et al., 1998; Owsianik et al., 2006; Ramsey et al., 2006; Tiruppathi et al., 2006; Moccia, 2012; Ong et al., 2016). TRPs can be activated by both intra- and extra-cellular messengers, as well as by physical or mechanical stimuli, promoting calcium signals and membrane depolarization, that may respectively, recruit store-operated and voltage-gated channels (Nilius, 2007; Mulier et al., 2017).

Consistent literature supports the involvement of TRP channels in both SOCE and non-SOCE calcium entry in ECs, as well as in endothelial permeability.

The molecular identity of the channels involved in endothelial SOCE gave rise to a long and exciting debate, with conflicting data due to methodological issues as culture conditions, overexpression systems, electrophysiological protocols, and pharmacological approaches (Groschner et al., 1998; Abdullaev et al., 2008; Trebak, 2009). The general accepted model recognizes highly Ca^2+^ selective SOCE currents through Orai1 channels and non-selective SOCE currents (the canonical I_*SOC*_) mediated by Orai1 and TRPC1, considered the predominant isoform expressed in human vascular endothelium (Tiruppathi et al., 2006; Worley et al., 2007; Cheng et al., 2013; Sabourin et al., 2015; Ambudkar et al., 2017; Lopez et al., 2020). The ER Ca^2+^ sensor STIM1 regulates both these kinds of channels (Lopez et al., 2020) and triggers Orai1 and TRPC1 activation by distinct C-terminus domains. Therefore, TRPC1 function is not only dependent on STIM1, but also requires the interaction with Orai1 (Ong et al., 2016). Moreover, Orai1-mediated Ca^2+^ entry is needed for recruitment of TRPC1 and its insertion into membranes, while STIM1 gates the channel (Cheng et al., 2013). In human ECs, the phosphorylation of TRPC1 by PKCa is essential for the thrombin-induced activation of SOCE (Ahmmed et al., 2004). The expression of TRPC1 is also regulated: in human PAECs (hPAECs), the inflammatory cytokine TNF-α promotes TRPC1 overexpression (Paria et al., 2004) that triggers Ca^2+^ influx and enhances endothelial permeability (Tiruppathi et al., 2006). Upon thrombin exposure, RhoA triggers the association of TRPC1 to InsP3R, its translocation to the plasma membrane, calcium entry enhancement and finally the increase in endothelial permeability (Mehta et al., 2003). A role for SPHK1 was suggested in the pathway by which TRPC1-mediated calcium entry destabilizes AJs: TRPC1 holds SPHK1 constitutively in a suppressed state to prevent SP1 production, enabling inflammatory agonists to mediate vascular leak (Tauseef et al., 2016; Simmons et al., 2019).

TRPC1 overexpression in hMEC caused a twofold increase in thrombin-induced calcium depletion and in InsP3 store-operated cationic current. Actin-stress fiber formation was augmented and TER decreased (Paria et al., 2004). On the contrary, TRPC1 depletion reduced the global cytosolic Ca^2+^ response by 25% and *I*_*SOC*_ by 50% (Brough et al., 2001). Further, the application of a specific antibody directed against an extracellular epitope of TRPC1 blocked thrombin- or InsP_3_-induced Ca^2+^ entry (Ahmmed et al., 2004).

Some members of TRPC family (i.e., TRPC-3, -6, and -7) can be stimulated by the membrane-permeant analog of DAG, 1-oleoyl-2-acetyl-sn-glycerol (Hofmann et al., 1999). Furthermore, these three channels show different levels of store-dependence: TRPC-3 is quite sensitive to InsP3-mediated responses, whereas TRPC-6 and -7 appear to be completely store-independent. Nevertheless, the independence of TRPC-3 from InsP3 receptor activation has been demonstrated in that PLC activation in InsP3 receptor-deficient cell lines still retained TRPC-3 activation (Tiruppathi et al., 2002; Pocock et al., 2004).

Beside TRPC1, TRPC4 was also proposed as a major contributor in SOCE (Wu et al., 2005; Trebak, 2009; Sundivakkam et al., 2012; Antigny et al., 2017). TRPC1 and TRPC4 can heterodimerize in ECs, forming a single functional channel (Antoniotti et al., 2006; Ma et al., 2011; Du et al., 2014; Greenberg et al., 2019). TRPC4 binds to STIM1 (Worley et al., 2007) as well as to Orai1: interestingly, Orai1 knockdown decreases the opening probability and the selectivity of TRPC1/4 channel (Cioffi et al., 2012; Thakore and Earley, 2019).

Knock out models for TRPC4 confirmed its role in permeability *in vivo*. Ca^2+^ influx evoked either by thrombin or a synthetic agonist (TFLLRNPNDK) was drastically diminished in isolated-perfused TRPC4-/- mouse lungs and in cultures ECs from the same model (Tiruppathi et al., 2002). This was associated with a lack of thrombin-induced actin-stress fiber formation and an impaired endothelial cell retraction.

Interestingly, TRPC4/5 channels can be mobilized not only *via* the Gq/11-protein–PLC pathway, but also following Gi/o-coupled signaling (Jeon et al., 2012).

Even if not demonstrated in ECs, recent evidences show that TRPC4/5 recruitment requires the dissociation of NHERF proteins from the channel C terminus, thus providing DAG sensitivity (Storch et al., 2017; Mederos y Schnitzler et al., 2018).

As stated above, TRPC6 may also be involved in EB dysfunction. Indeed, in frog mesenteric microvessels, VEGF-induced increase in vascular permeability can be mimicked by DAG, an agonist of TRPC3/6/7. Furthermore, flufenamic acid, which positively regulates TRPC6 but inhibits -C3 and -C7, enhances the effect exerted by VEGF (Pocock et al., 2004).

Evidences from studies with transfected cells demonstrated the TRPCs can mediate Ca^2+^ gated by DAG and are store and PKC independent (Nilius and Droogmans, 2001). Consistently, OAG-induced Ca^2+^ currents in TRPC-6-expressing cells were not sensitive to PKC inhibition, suggesting that TRPC-6 is directly gated by DAG (Hofmann et al., 1999).

Moreover, in Pocock et al. (2004) demonstrated *in vivo* that at least one mechanism of action of VEGF involves the increase in [Ca^2+^]_i_ through store-independent TRPC-6 activation.

Finally, by the use of calcium imaging in isolated perfused rat lungs and patch clamp in rPAECs, TRPC6 was found to be critically involved in lung vascular leakage after stimulation with platelet-activating factor through its recruitment into caveolae (Samapati et al., 2012). In human PAECs, cell contraction exerted by thrombin is mediated by TRPC6 *via* a PKA-dependent pathway (Singh et al., 2007).

A member of TRPM subfamily, TRPM2, is highly expressed in ECs (Simmons et al., 2019), but its role in permeability is controversial. In cultured hPAECs, TRPM2 is opened by intracellular ADP-ribose and mediates the hyperpermeability triggered upon exposure to H_2_O_2_. Some *in vivo* studies reported that TRPM2^–/–^ mice develop pulmonary edema upon LPS treatment (Thomas et al., 2012), but other authors failed to reproduce the evidence (Hardaker et al., 2012). The discrepancy could be due to different mouse strains used in the experiments (Thakore and Earley, 2019).

In the ECs of blood vessels following spinal cord injury, TRPM4 resulted up-regulated, but the underlying mechanism in vascular permeability remains unclear (Grunewald et al., 2006).

Finally, some components of TRPV subfamily are under intense investigation.

The role of TRPV1 channels in this context is controversial. Indeed, Alvarez et al. demonstrated that the TRPV1 agonists did not affect lung permeability (Alvarez et al., 2005). Nevertheless, Wang et al. reported a protective effect by capsaicin, a potent TRPV1 activator, in rabbit lung ischemia-reperfusion injury (Wang et al., 2012; Thakore and Earley, 2019). On the other hand, TRPV1 expression is very low in hMECs (Thomas et al., 2012).

Nowadays, TRPV4 is attracting much attention for its influence on capillary barrier function (Morty and Kuebler, 2014; Simmons et al., 2019). Downregulation of TRPC1 and TRPC4 in artery and vein lung endothelium is associated with the loss of response to the SOCE-activating compound thapsigargin, supporting the idea that thapsigargin triggers a TRPC1/TRPC4-containing channel to increase permeability. However, permeability regulated by EET, a metabolite of the AA, was retained, opening the possibility for the involvement of other channels (Alvarez et al., 2005). 14,15-EET, which is produced upon high peak inspiratory pressure, activates TRPV4 and the subsequent increase in pulmonary vascular permeability (Watanabe et al., 2002; Hamanaka et al., 2007; Simmons et al., 2019; Thakore and Earley, 2019). TRPV4 is abundantly expressed in the endothelium from lung intra-alveolar capillaries and, to a lesser extent, from large, extra-alveolar vessels (Danbara et al., 1982; Alvarez et al., 2006). The TRPV4 agonist 4αPDD increased permeability in wild type capillaries, with disruption of cell-matrix tethering, but failed to exert any effect in TRPV4 knockout mice; on the other hand, thapsigargin produced the same effect in extra-alveolar vessels (Alvarez et al., 2006; Thakore and Earley, 2019). Therefore, TRPC1 and TRPC4 appear to interfere preferentially with the lung extra-alveolar EB, whereas TRPV4 is prominently functional in lung capillaries (Cioffi et al., 2009). In mice, TRPV4 knockout prevented the permeability increase of lung microvasculature induced by both 4αPDD and 14,15-EET, without affecting SOCE (Komarova et al., 2017). Moreover, TRPV4 triggers Ca^2+^-activated K^+^ channels (KCa) in rPMECs, enhancing the driving force for calcium entry and the response to TRPV4 (Lin et al., 2015). In mouse and human LMECs, ROS enhance vessel permeability *via* a Fyn Src kinase-TRPV4-dependent Ca^2+^ influx (Suresh et al., 2015; Thakore and Earley, 2019). TRPV4 seems to be also involved in the pulmonary edema associated with heart failure (Thorneloe et al., 2012). Interestingly, a NO/cGMP-dependent negative feedback loop was discovered for protection against the excessive microvascular barrier permeability (Yin et al., 2008). In LMECs, mechanically gated recruitment of TRPV4 elicits the release of the MMP MMP2 and MMP9, which degrade collagen IV and laminin, key structural components of the alveolar basement membrane, as well as integrins, intercellular E-cadherin and other intercellular targets (Villalta et al., 2014). The overall effect is the endothelial detachment from the basement membrane (Willette et al., 2008; Villalta and Townsley, 2013) and a permeability enhancement.

TRPV4 is also expressed in retinal hMECs and in intact blood vessels of the inner retina. Its selective agonist GSK101 promoted MEC permeability in association with disrupted F-actin organization, occludin downregulation and adherent contacts remodeling. Moreover, GSK101 increased the permeability of WT retinal blood vessels, but not in TRPV4 knockout mice, pointing to a major role for the channel in Ca^2+^ homeostasis and retinal barrier function (Phuong et al., 2017).

## Conclusion

**FIGURE 1:**
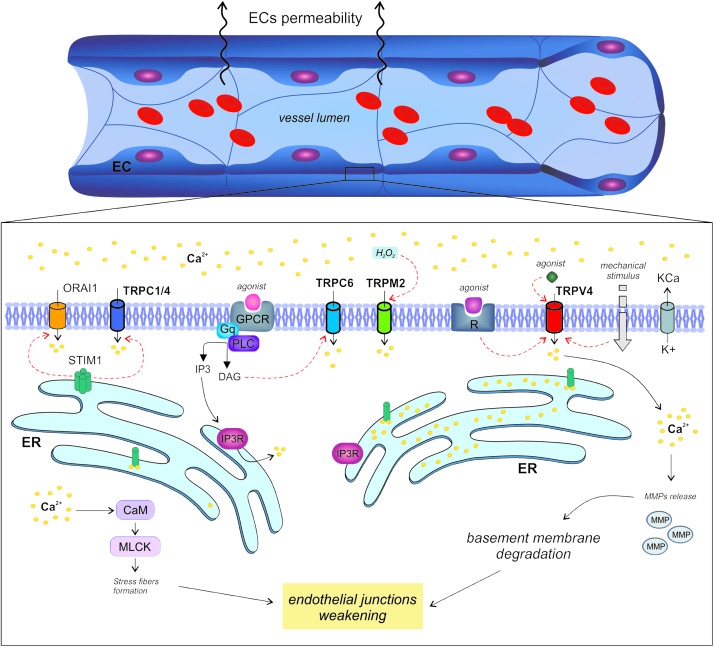
Regulation of endothelial permeability by TRP channels. The endothelial calcium signals regulate vascular permeability in large vessels as well as in capillaries through different mechanisms.

Calcium signaling tunes endothelial permeability in capillaries as well as in larger vessels. However, it is not surprising to find a huge variability in the related molecular machinery due to the well known heterogeneity of the endothelium in different tissues (Figure 1).

A general and established model for the relative contribution of SOCE and non-SOCE in macro- and micro-vessel permeability, as well as for the identity and function of TRP channels, is not available yet. A severe limitation is the lack of a detailed TRP proteomic pattern and intracellular targeting landscape in capillary and arterial/vein ECs. In addition, selective pharmacological compounds are only available for some TRP members and some of them are not suitable for systemic treatments due to their highly toxicity (Meotti et al., 2014; Rodrigues et al., 2016; Rubaiy et al., 2017; Lawhorn et al., 2020).

Finally, the simple detection of a variety of active calcium entry mechanisms, with their complex protein machineries, does not necessarily provide an evidence for their exclusive functional involvement in vessel permeability. Trebak and coworkers reported that the acute barrier disruption activated by thrombin in hUVECs and hMECs requires endoplasmic-reticulum localized STIM1 independently of Orai1, MLCK, and Ca^2+^ entry across the plasma membrane. STIM1 couples the thrombin receptor, recruits guanosine triphosphatase RhoA and stimulates MLC phosphorylation, finally leading to formation of actin stress fibers and loss of cell-cell adhesion (Shinde et al., 2013; Stolwijk et al., 2016).

Vascular permeability is probably the result of a concurrence among diverse, tissue-dependent intracellular processes, all contributing in variable weights to the overall event.

## Author Contributions

All authors listed have made a substantial, direct and intellectual contribution to the work, and approved it for publication.

## Conflict of Interest

The authors declare that the research was conducted in the absence of any commercial or financial relationships that could be construed as a potential conflict of interest.

## Abbreviations

4 α PDD: 4 α-phorbol 12,13-didecanoate
AA: arachidonic acid
AJs: adherent junction complexes
EB: endothelial barrier
ECM: extracellular matrix
ECs: endothelial cells
EET: 14,15-epoxyeicosatrienoic acid
FACs: focal adhesion complexes
hMEC: human microvascular endothelial cells
IEJs: intercellular endothelial junctions
LMECs: lung microvascular EC
MLC: Myosin light chain
MMP: matrix metalloproteinases
nSOCs: non-store-operated channels
ROS: reactive oxygen species
rPAECs: rat pulmonary aortic endothelial cells
rPMECs: rat pulmonary microvascular endothelial cells
SOCE: Store-Operated Calcium Entry
SOCs: store-operated cation channels
SP1: sphingosine-1-phosphate
SPHK1: sphingosine kinase 1
TER: transendothelial electrical resistance
TNF- α: tumour necrosis factor- α
TRP: Transient receptor potential.
